# Selenocysteine modulates resistance to environmental stress and confers anti-aging effects in *C. elegans*

**DOI:** 10.6061/clinics/2017(08)07

**Published:** 2017-08

**Authors:** Jun-Sung Kim, So-Hyeon Kim, Sang-Kyu Park

**Affiliations:** Soonchunhyang University, College of Medical Sciences, Department of Medical Biotechnology, Asan, Chungnam, Republic of Korea

**Keywords:** Selenocysteine, Stress Response, Lifespan, Aging, *C. elegans*

## Abstract

**OBJECTIVE:**

The free radical theory of aging suggests that cellular oxidative damage caused by free radicals is a leading cause of aging. In the present study, we examined the effects of a well-known anti-oxidant amino acid derivative, selenocysteine, in response to environmental stress and aging using *Caenorhabditis elegans* as a model system.

**METHOD:**

The response to oxidative stress induced by H_2_O_2_ or ultraviolet irradiation was compared between the untreated control and selenocysteine-treated groups. The effect of selenocysteine on lifespan and fertility was then determined. To examine the effect of selenocysteine on muscle aging, we monitored the change in motility with aging in both the untreated control and selenocysteine-treated groups.

**RESULTS:**

Dietary supplementation with selenocysteine significantly increased resistance to oxidative stress. Survival after ultraviolet irradiation was also increased by supplementation with selenocysteine. Treatment with selenocysteine confers a longevity phenotype without an accompanying reduction in fertility, which is frequently observed in lifespan-extending interventions as a trade-off in *C. elegans*. In addition, the age-related decline in motility was significantly delayed by supplementation of selenocysteine.

**CONCLUSION:**

These findings suggest that dietary supplementation of selenocysteine can modulate response to stressors and lead to lifespan extension, thus supporting the free radical theory of aging.

## INTRODUCTION

Aging is one of the most complex biological pathways, with hundreds of theories attempting to explain the aging process. The leading theory is the free radical theory of aging, which suggests that the accumulation of oxidative damage to cellular macromolecules caused by free radicals is the major cause of normal aging 1. The mitochondrial decline theory of aging emphasizes the function of the mitochondria in aging. The theory is based on the fact that the mitochondrion is the most powerful free radical-producing organelle in the cell through its electron transport chain reaction 2. Other well-known theories of aging include the genomic instability theory, Hayflick limit theory, telomerase theory, and membrane theory 3, 4. Despite these various theories, there is no single theory of aging that can explain all phenomena observed in the aging process. Therefore, people believe that the various theories of aging are closely inter-related 4.

Based on the free radical theory of aging, the effects of anti-oxidants that can ameliorate cellular oxidative damage on lifespan and age-related alterations have been studied. Dietary supplementation with resveratrol, an anti-oxidant abundant in red wine, increases lifespan and age-related physiological changes in many model organisms 5, 6. Resveratrol also has a positive effect in ameliorating many age-related diseases, including cancer and Alzheimer’s disease 7, 8. Another well-known anti-oxidant, vitamin E, also extends lifespan and retards age-related transcriptional changes in the brain and muscles of mice 9. In *Caenorhabditis elegans*, animals grown in media prepared with electrolyzed-reduced water have shown an extended lifespan compared with animals grown in media prepared with distilled water 10. Electrolyzed-reduced water has been shown to have anti-oxidant activity 11. A recent study demonstrated that N-acetyl-L-cysteine, a cysteine derivative exhibiting strong anti-oxidant activity, confers a longevity phenotype and increased resistance to environmental stressors 12. N-acetyl-L-cysteine induces the expression of *hsp-16.2* and *sod-3*, which are positively correlated with an individual’s lifespan in *C. elegans* 12.

Selenocysteine is a cysteine derivative containing selenium 13. Selenium is known for its anti-cancer and anti-inflammatory properties 14, 15. Selenocysteine is incorporated into various anti-oxidant enzymes, including glutathione peroxidase and thioredoxin reductase, and acts as the active site in the cellular anti-oxidant defense system 16. Selenium deficiency is associated with many diseases, including cancer, cardiovascular disease, and osteoarthritis 17. In contrast, excess selenium generates reactive oxygen species (ROS) and triggers apoptotic cell death 17. *In vitro* analysis reveals that selenocysteine has a lower pKa than cysteine, which can create an acidic environment 18. In aged rats, selenium reduces oxidative stress, apoptosis, and memory impairment 19. Synthetic oligopeptides containing selenocysteine decrease the production of reactive oxygen species and suppress apoptosis through the regulation of the Bcl-2/Bax ratio 20. In humans, mutations in selenocysteine synthase, an enzyme catalyzing the synthesis of selenocysteine, cause early-onset neurological damages, such as cerebellar atrophy 21. In contrast, *Drosophila* mutants deficient in the biosynthesis of selenocysteine exhibit normal viability, lifespan, and response to oxidative stress 22. These findings suggest that the effect of selenocysteine may be species-specific. Selenocysteine-containing thioredoxin reductase is required for molting, the removal of old cuticle from the epidermis of *C. elegans* 23. Thioredoxin reductase naturally decreases with aging 23. Treatment with the selenium-containing xylofuranoside, a compound synthesized from D-xylose, reduces Mn-induced toxicity in *C. elegans* 24. Xylofuranoside also induces the up-regulation of *sod-3* and the nuclear localization of DAF-16, a transcription factor involved in stress response and aging in *C. elegans* 24.

In the present study, we examined the effect of selenocysteine in response to environmental stressors and aging. The change in resistance to oxidative stress induced by hydrogen peroxide by selenocysteine supplementation was monitored using *C. elegans* as the model system. The survival of worms after ultraviolet (UV) irradiation was used to compare untreated and selenocysteine-treated worms. The effect of supplementation with selenocysteine on the organism’s lifespan and reproductive capabilities was measured *in vivo*. We also investigated the effect of selenocysteine on the age-related decline of motility, one of the age-related physiological changes observed in *C. elegans*.

## MATERIALS AND METHODS

### Worm strains and culture

The N2 CGCb strain of *C. elegans*, purchased from the *C. elegans* Genetics Center (CGC, Minneapolis, USA), was used as the wild-type control. Solid nematode growth media (NGM) plates containing 25 mM NaCl, 1.7% agar, 2.5 mg/mL peptone, 5 μg/mL cholesterol, 1 mM CaCl_2_, 1 mM MgSO_4_, and 50 mM KH_2_PO_4_ (pH6.0) were used as the growth media. All experiments were conducted at 20°C. *Escherichia coli* OP50 was added to each NGM plate as a source of food.

### Survival under oxidative stress

Five L4/young adult worms were transferred to a fresh NGM plate and permitted to lay eggs for 5 h. After removing the five adult worms, the progeny were grown on NGM plates for 3 days. Age-synchronized worms were treated with different concentrations (0, 1, 2.5, or 5 mM) of selenocysteine (Sigma-Aldrich, St. Louis, USA) for 24 h. The worms were then exposed to 1 or 2 mM H_2_O_2_ in S-basal without cholesterol (5.85 g sodium chloride, 1 g potassium phosphate dibasic, and 6 g potassium phosphate monobasic for 1 L sterilized distilled water). The survival of worms under oxidative-stress conditions was monitored after 6 h. Worms not responding to any mechanical stimuli were considered dead. Three independent replicate experiments were performed. Statistical significance was measured using the standard two-tailed Student’s t-test. *P*-values less than 0.05 were considered significant.

### Resistance to UV irradiation

Age-synchronized worms were cultured in NGM plates containing different concentrations of selenocysteine (0, 1, 2.5, and 5 mM) for 24 h and exposed to UV light (20 J/cm^2^/min) for 1 min using a 254 nm UV crosslinker (BLX-254, VILBER Lourmat Co., Torcy, France). After UV irradiation, the plates were transferred back to the 20°C incubator. Living and dead worms were scored every day until all worms were dead. For statistical analysis, we employed the log-rank test 25.

### Lifespan assay

Sixty age-synchronized 3-day-old worms were transferred to fresh NGM plates containing different concentrations of selenocysteine (0, 1, 2.5, or 5 mM). 5-Fluoro-2ʹ-deoxyruridine (12.5 mg/L) was added to prevent internal hatching. Thereafter, worms were transferred to fresh NGM plates containing different concentrations of selenocysteine and 12.5 mg/L of 5-fluoro-2ʹ-deoxyruridine every other day until all worms were dead. The number of living and dead worms was scored every day. The log-rank test was used for statistical analysis 25.

### Fertility assay

Five L4/young adult worms were transferred to a fresh NGM plate containing different concentrations of selenocysteine (0, 1, 2.5, or 5 mM) and permitted to lay eggs for 5 h. The eggs were maintained at 20°C for 2 days. Ten 2-day-old worms were transferred to 10 fresh NGM plates individually containing different concentrations of selenocysteine every day until each worm laid no eggs. Eggs spawned on each day by an individual worm were incubated at 20°C for 48 h, and the number of progeny produced was recorded. The average number of progeny produced by 10 individual worms treated with different concentrations of selenocysteine was compared with that of the control by ANOVA.

### Locomotion assay

The effects of selenocysteine on the age-related decline in motility was monitored using age-synchronized worms (n=100). Each worm’s response to mechanical stimuli was classified into three levels. Worms that moved spontaneously without mechanical stimuli were labeled “phase 1”. Worms that moved their whole body or solely their head after worm picker stimulation were labeled “phase 2” or “phase 3”, respectively. Each worm’s response to mechanical stimuli was recorded at days 5, 10, 15 and 20 after hatching.

## RESULTS

### Effect of selenocysteine on survival under oxidative stress induced by H_2_O_2_

**Figure 1:**
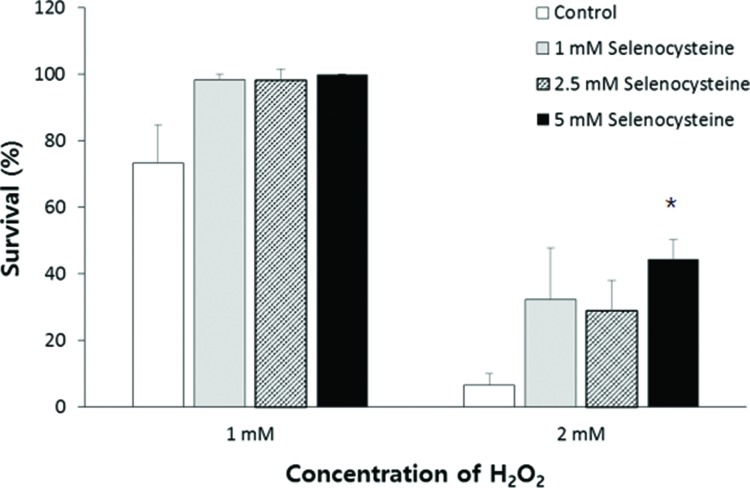
Increased resistance to oxidative stress conferred by selenocysteine. Sixty age-synchronized young adult worms were pre-treated with different concentrations of selenocysteine for 24 h, then transferred to S-basal containing 1 or 2 mM H_2_O_2_ to induce oxidative stress. After 6 h, the survival of worms was recoreded. Data indicate mean survival of three independent experiments. Asterisks indicate *p*-values less than 0.05 compared with the untreated control.

To determine the effect of selenocysteine in response to oxidative stress, we compared the survival of worms under oxidative stress between the control group and the experimental groups pre-treated with different concentrations of selenocysteine. Supplementation with selenocysteine failed to show a significant difference in the survival of worms incubated with 1 mM H_2_O_2_. The percent survival in the untreated control was 73.3±11.63% (mean±SEM), and that in the selenocysteine-treated groups was greater than 98%; the values were not significantly different (0.05<*p*<0.1). When the authors used a higher concentration of H_2_O_2_ (2 mM) to induce oxidative stress, supplementation with selenocysteine resulted in a significant difference in survival between the control and selenocysteine-treated groups. Only 6.7±3.33% of worms survived in the untreated control groups, whereas 28.9±9.09 (*p*=0.083) and 44.4±5.88% (*p*=0.005) of worms survived in the experimental groups pre-treated with 2.5 and 5 mM selenocysteine, respectively (Figure 1).

### Effect of selenocysteine on survival after UV irradiation

**Figure 2:**
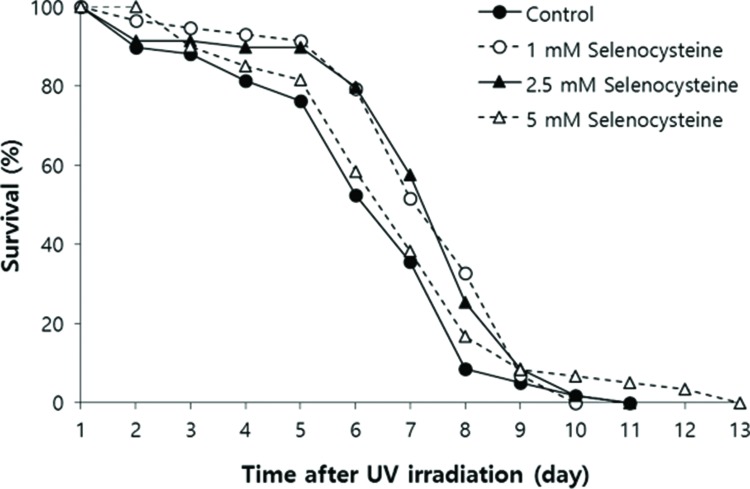
Effects of selenocysteine on survival after UV irradiation. After 24 h of selenocysteine pre-treatment, animals were irradiated with 20 J/cm^2^/min of UV light for 1 min. The time-course % survival of age-synchronized worms (n=60) was then monitored every day until all worms were dead in the untreated and selenocysteine-treated groups. Mean survival time was significantly increased with 1 or 2.5 mM selenocysteine (*p*<0.05).

Next, we examined the effect of selenocysteine on survival after UV irradiation. As shown in Figure 2, the mean survival time of the untreated control group was 6.39 days. In the selenocysteine-treated groups, the mean survival times were extended by up to 7.36 days with 1 or 2.5 mM selenocysteine (15.1% increase, *p*<0.05). Unlike the effect on the response to oxidative stress, 5 mM selenocysteine failed to show a significant effect on the survival rate and time after UV irradiation. The mean survival time was 6.93 days, which was not statistically significantly different from that of the control (*p*=0.302).

### Lifespan-modulating effect of selenocysteine in *C. elegans*

**Figure 3:**
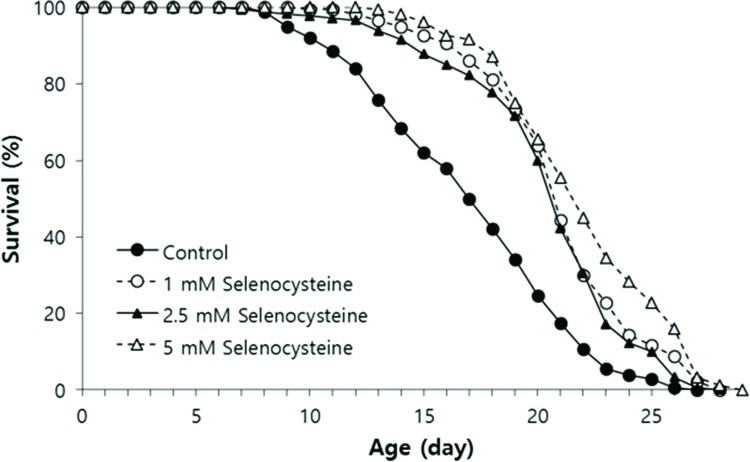
Lifespan-extending effect of selenocysteine. Age-synchronized young adult worms (n=60) were transferred to NGM plates containing differenct concentrations of selenocysteine, and the number of alive/dead worms was recorded every day. Worms that were lost or showed internal hatching during the assay were excluded. For all concentrations of selenocysteine tested, the mean lifespan was markedly increased compared with that of the untreated control (*p*<0.05).

We tested the effect of selenocysteine on lifespan in *C. elegans*. In the first experiment, the mean lifespan was increased from 16.3 days in the untreated control group to 20.9 days (27.6% increase) with 1 mM selenocysteine, 20.6 days (25.9% increase) with 2.5 mM selenocysteine, and 22.2 days (35.9% increase) with 5 mM selenocysteine (Table 1). The maximum lifespan was also increased from 24 days to 27 days with 5 mM selenocysteine (Figure 3). Independent repeated experiments showed the same significant increase in lifespan with all concentrations of selenocysteine tested. The survival curve shown in Figure 3 was drawn using the average values of three independent lifespan assays.

### Impact on reproduction by selenocysteine in *C. elegans*

**Figure 4:**
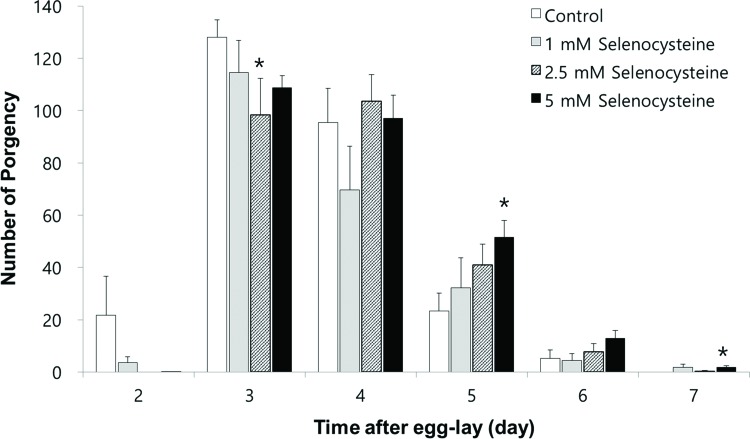
Effects of selenocysteine on reproduction. The number of progeny produced was monitored every day during a gravid period. Data show the mean number of progeny produced by 10 individual worms on each day. Asterisks indicate *p*-values less than 0.05 compared with the untreated control.

Next, we examined the effect of selenocysteine on organisms’ reproduction. As shown in Figure 4, the gravid period was shifted by selenocysteine supplementation. The untreated control worms produced progeny from day 2 to day 6 after hatching. However, the worms treated with selenocysteine produced small amounts of progeny on day 2 and maintained fertility until day 7 after hatching. Among the different concentrations of selenocysteine tested, 5 mM of selenocysteine caused a significant difference in the number of progeny produced compared with the untreated control. On day 2, 21.7±13.14 (mean±SEM) progeny were produced by the untreated control, whereas only 0.1±0.13 progeny were produced by the worms treated with 5 mM selenocysteine. The numbers of progeny produced on day 3 were 128.1±5.82 and 108.8±4.62 in the untreated control and the 5 mM selenocysteine-treated groups, respectively (*p*=0.098). However, more progeny were produced at a later stage of the gravid period by the worms treated with 5 mM selenocysteine. The number of progeny produced was increased from 23.3±6.17 in the untreated control to 51.5±6.49 in the worms treated with 5 mM selenocysteine on day 5 (*p*=0.031). The worms treated with 5 mM selenocysteine continued to produce 1.8±0.59 progeny, whereas no progeny were produced by the untreated control on day 7 (*p*=0.049) (Figure 4). The total number of progeny produced during the gravid period was not significantly affected by the supplementation with selenocysteine.

### Effect of selenocysteine on age-related decline in motility

**Figure 5:**
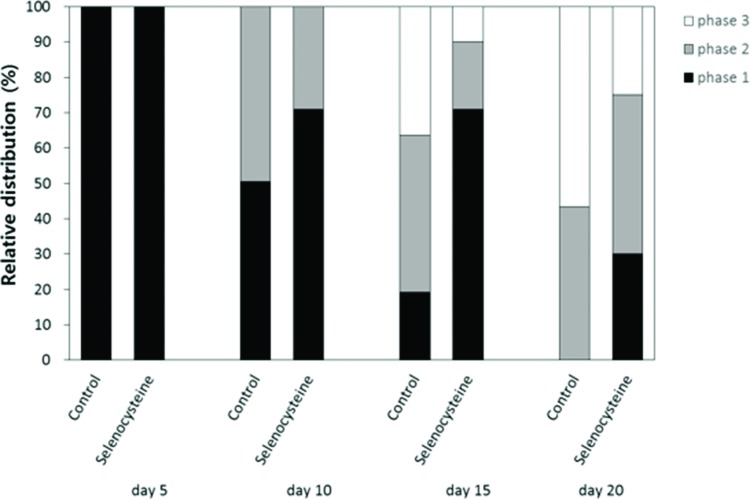
Delayed age-related decline in motility by selenocysteine. The relative distribution of worms in different locomotive phases was determined on the indicated days for the untreated control and 5 mM selenocysteine-treated animals. ■ phase 1, worms moved spontaneously without any stimuli. 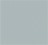 phase 2, worms moved whole body in response to mechanical stimuli. □ phase 3, worms moved only head in response to mechanical stimuli.

To examine the effect of selenocysteine supplementation on muscle aging, we monitored changes in motility over time in worms untreated and treated with selenocysteine. The age-related decline in motility was retarded by supplementation with selenocysteine (Figure 5). Ten days after hatching, more worms were classified as “phase 1”, including worms that could move spontaneously without any mechanical stimuli in the selenocysteine-treated group, compared with the untreated control. At 10 days of age, 50.5% of animals were classified as “phase 1” in the untreated control and 71.0% of the selenocysteine-treated worms classified as “phase 1”. On day 15, only 19.2% of the worms were classified as “phase 1” in the control, whereas 71.0% of worms were still classified as “phase 1” in the selenocysteine-treated group. In contrast, the number of worms that could move only their head after stimulation, “phase 3”, was less in the selenocysteine-treated group than in the untreated control. The percentage of worms classified as “phase 3” was decreased from 36.4% in the untreated control worms to 10.0% by the supplementation with selenocysteine. Thirty percent of the selenocysteine-treated worms were still able to move freely without any stimuli (phase 1), whereas no worm was classified as “phase 1” in the untreated control on day 20 (Figure 5).

## DISCUSSION

A positive correlation between increased resistance to environmental stressors and lifespan extension was observed with numerous genetic and nutritional interventions. One of the most conserved age-modulating cellular pathways among various organisms, from yeasts to humans, is insulin/IGF-1-like signaling 26. Mutations in *daf-2*, a receptor for insulin/IGF-1-like ligand, and *age-1*, an intracellular adaptor molecule involved in insulin/IGF-1-like signaling, lead to an extended lifespan in *C. elegans* 27. Long-living *daf-2* or *age-1* mutants also exhibit increased survival after exposure to environmental stressors, including oxidative stress, heat shock, and UV irradiation 28. Dietary restriction confers the longevity phenotype in *C. elegans*, *Drosophila melanogaster*, and mice 29, 30. Dietary-restricted animals show increased resistance to oxidative stress and reduced incidence of a number of age-related diseases 31. Supplementation with anti-oxidants, such as resveratrol, N-acetyl-L-cysteine, and curcumin, causes an increase in both lifespan and resistance to stressors in *C. elegans* 6, 12, 32. Extracts from *Acanthpanax sessiliflorus* have both anti-oxidant and lifespan-extending properties *in vivo* 33. In mice, the effect of anti-oxidants on lifespan is still controversial. For example, vitamin E supplementation results in increased lifespan, whereas supplementation with coenzyme Q_10_ or lycopene fails to produce a longevity phenotype, despite reducing the incidence of tumors 34, 35. In the present study, we observed increased survival under oxidative stress and UV irradiation via supplementation of selenocysteine. These findings suggest that dietary supplementation with selenocysteine can increase resistance to environmental stresses in a dose-dependent manner in *C. elegans* and provide evidence for the *in vivo* anti-stress activity of selenocysteine. Having observed increased resistance to oxidative stress and UV irradiation by selenocysteine, the authors asked whether dietary supplementation with selenocysteine could affect the lifespan of *C. elegans*. A lifespan assay revealed that selenocysteine can in fact significantly extend both mean and maximum lifespan in *C. elegans*. Our findings indicate that dietary supplementation with selenocysteine does confer a longevity phenotype in *C. elegans*, possibly by modulating the response to environmental stressors, supporting the free radical theory of aging. Future studies should focus on the effect of selenocysteine on ROS level and the activity of anti-oxidant enzymes, the identification of the underlying cellular mechanisms involved, and the relationship with other known lifespan-extending genetic pathways.

The disposable soma theory of aging was first hypothesized by Thomas Kirkwood in 1977 and suggested that an organism should distribute limited cellular resources to reproductive ability and the maintenance of somatic cells 36. Previous studies have shown that numerous mutants with extended lifespan exhibit reduced fertility or a delayed gravid period in *C. elegans*, which suggests that reduced reproductive activity is a natural trade-off of lifespan extension 37, 38. For example, long-living *daf-2* mutants have exhibited reduced fertility 37. A number of lifespan-extending dietary interventions also accompany reduced fertility and/or a delayed gravid period in *C. elegans*. Dietary supplementation with the anti-oxidant resveratrol increases lifespan and reduces fecundity 39. Complete knockout of germ cells also increases lifespan in *C. elegans* 40. In contrast, dietary supplementation with N-acetyl-L-cysteine confers a longevity phenotype and enhances fertility 12. Here, we demonstrated that the total number of progeny produced during a gravid period was not affected by selenocysteine. However, a delay of the gravid period in worms treated with 5 mM selenocysteine was observed. These results indicate that the lifespan-extending effects of selenocysteine accompany a delayed gravid period as a possible trade-off for producing the longevity phenotype. Another study showed that polyphenols from blueberries extended lifespan and caused a delay in the decline of pumping rate as a trade-off, suggesting that there can be alternative trade-offs for lifespan extension 41.

Muscle tissue demands high amounts of ATP produced by mitochondria for proper functioning and is susceptible to ROS produced by mitochondria as a byproduct during ATP generation. The loss of muscle mass and strength is defined as sarcopenia 42. Recent studies suggest that age-related degeneration of muscle is associated with age-related dysfunction of mitochondria, which generate less ATP and produce more free radicals 43, 44. Anti-oxidant *Sod-1*-deficient mice show a premature aging phenotype and early-onset sarcopenia 45. In contrast, over-expression of mitochondrial catalase, an anti-oxidant enzyme, reduces the accumulation of oxidative damage and delays age-related decline of muscle function in mice 46. In *C. elegans*, an individual’s motility declines with age. Therefore, the age-related decline of motility is one of the most widely used biomarkers for aging in *C. elegans*. Dietary supplementation with silymarin, a flavanone derivative found in milk thistle (*Silybum marianum*), extends lifespan and increases locomotion rate in *C. elegans* 47. Silymarin also markedly protects amyloid beta-induced toxicity expressed in muscle 47. Phycoerythin, a strong anti-oxidant isolated from marine cyanobacteria, confers a longevity phenotype and enhances indicators of health, including pharyngeal pumping and locomotion rate 48. Our study showed that dietary supplementation with selenocysteine significantly delayed the age-related decline of motility in *C. elegans*. This result suggests that selenocysteine has an anti-aging effect on muscle tissue. Future studies should determine the underlying mechanisms involved in the effect of selenocysteine.

Hormesis is defined as the beneficial response to the exposure of a low dose of harmful interventions. In *C. elegans*, increased resistance to stress and extended lifespan have been observed due to the hormesis effect of free radicals, heat stress, and dietary restriction (DR) 49. The effect of DR in particular has been reported in various model organisms 50. DR increases resistance to stressors and extends lifespan in yeasts, worms, flies, and mice 50. Supplementation with N-acetyl-L-cysteine increases resistance to oxidative stress at low doses and decreases resistance to oxidative stress at high doses, which suggests a possible hormesis effect of N-acetyl-L-cysteine 12. Because both beneficial and harmful effects of selenocysteine have been reported, our observations could be due to a hormesis effect. Further studies should focus on the identification of cellular pathways involved in selenocysteine’s effects to fully understand the *in vivo* activity of selenocysteine.

The longevity phenotype observed in this study using *C. elegans* cannot be directly carried over to higher organisms. Therefore, a study of the effect of selenocysteine in other model organisms should follow. In addition, the effect of selenocysteine on age-related disorders will be useful for practical applications of selenocysteine.

## AUTHOR CONTRIBUTIONS

Park SK conceived and designed the study and reviewed the manuscript. Kim JS performed and analyzed all experiments and wrote the manuscript. Kim SH performed repeated experiments for stress response and provided a critical review of the manuscript.

## Figures and Tables

**Table 1:** Effect of selenocysteine on lifespan in *C. elegans.*

	Selenocysteine (mM)	Mean lifespan (day)	Maximum lifespan (day)	*p*-value^1^	% effect^2^
1^st^ experiment	0	16.3	24		
	1	20.9	26	<0.001	27.6
	2.5	20.6	25	<0.001	25.9
	5	22.2	27	<0.001	35.9
2^nd^ experiment	0	17.0	28		
	1	23.9	29	<0.001	33.3
	2.5	21.9	28	0.002	21.7
	5	22.6	28	<0.001	25.8
3^rd^ experiment	0	20.1	24		
	1	21.5	27	0.003	7.0
	2.5	22.2	29	<0.001	10.3
	5	21.6	29	0.010	7.2

1 *p*-values were calculated using the log-rank test by comparing the survival of the untreated control group (0 mM selenocysteine) with that of the experimental groups treated with different concentrations of selenocysteine.

2 % effects were calculated by (*C*-*S*)/C*100, where *S* is the mean survival time of the experimental groups and *C* is the mean survival time of the untreated control group.
